# Development of novel oncology biomarkers for cancer

**DOI:** 10.1186/1753-6561-8-S4-O16

**Published:** 2014-10-01

**Authors:** Nathan Yoganathan

**Affiliations:** 1 KalGene Pharmaceuticals Inc, Canada

## Introduction

According to American Cancer Society projections for 2014, approximately 1.5 million new cancers are expected to be diagnosed and more than several hundred patients are expected to die of cancer, with more than 90% of these cancers being solid tumours (ACS, 2010). Epithelial cell-derived cancers comprise approximately 80-85% of all cancers, and include, amongst others, breast, bladder, lung, pancreatic, thyroid and prostate cancers [1-4]. Presently, breast cancer is the most common cancer among women worldwide as more than 1 in 4 cancers in women (about 28%) are of the breast, and thyroid cancer is the most common epithelial cell-derived malignancy of the endocrine glands [4,5]. These cancers may exhibit no signs in its early stages. Further, the aggressive cases are difficult to detect and when undetected, prognosis deteriorates rapidly.

Neutralization antibodies targeted to genes associated with cancer is a good strategy to detect cancer at early stages among many treatment approaches because of its high specificity and affinity. KalGene is developing a multi-protein assay to identify aggressive epithelial cancers, including breast, colon, and prostate, thyroid cancers. We have successfully developed several monoclonal antibodies against important biomarkers. Our monoclonal antibodies have shown excellent affinity and specific reactivity to the recombinant protein and cell lysates.

Purified monoclonal antibodies were tested on tissue microarrays (TMA). Tissue microarray technology allows a massive acceleration of studies correlating molecular *in situ* bindings with clinico-pathological information. In this method, minute tissue cylinders (diameter 0.6 mm) are removed from hundreds of different primary tumour blocks and subsequently brought into one empty 'recipient' paraffin block. Sections from TMA blocks can be used for all different types of *in situ* tissue analyses including immunohistochemistry. TMA sections were de-paraffinized and heat-mediated antigen retrieval was performed. Non-specific immunoglobulin binding was blocked with 10% normal horse serum (NHS) for 30 min at room temperature. The TMA sections were then incubated with the appropriate diluted Kalgene monoclonal antibody in NHS. Thereafter, the slides were incubated with the appropriate secondary antibody diluted in NHS. Signal from the secondary antibody can be detected, then visualized.

## Results and conclusions

The membranous KAL001 staining was calculated as a weighted average, based on results from three core samples per tumour. KAL001 expression levels were positively associated with well (versus poorly) differentiated tumours (n = 18; p = 0.05), low preoperative serum carcinoembryonic antigen (n = 76; p = 0.0002), and 5 year survival (n = 128; p = 0.01). The presence of perineural invasion and macroperforation were associated with lower KAL001 staining scores, but small sample numbers precluded statistical analysis of these results. Our study findings demonstrate that reduced KAL001
